# Diagnostic Value of Telomerase Activity in Patients With Bladder Cancer: A Meta-Analysis of Diagnostic Test

**DOI:** 10.3389/fonc.2020.570127

**Published:** 2020-12-03

**Authors:** Lei Peng, Jinze Li, Chunyang Meng, Jinming Li, Dandan Tang, Fangxue Guan, Peng Xu, Tangqiang Wei, Yunxiang Li

**Affiliations:** ^1^ Department of Urology, Nanchong Central Hospital, The Second Clinical College, North Sichuan Medical College (University), Nanchong, China; ^2^ Department of Urology, The Affiliated Hospital of Medical College, North Sichuan Medical College (University), Nanchong, China; ^3^ Department of Cardiothoracic Surgery, Shenzhen People’s Hospital, Affiliated Hospital of Jinan University, Shenzhen, China; ^4^ Internal Medicine, People’s Hospital of Yanyuan, Xichang City, China; ^5^ Department of Cardiology, The Affiliated Hospital of Medical University, Guizhou Medical University, Guizhou, China

**Keywords:** bladder cancer, telomerase activity, meta-analysis, diagnosis, biomarker

## Abstract

**Background:**

This study aimed to evaluate the diagnostic value of telomerase activity (TA) for bladder cancer (BC) by meta-analysis.

**Methods:**

We conducted a systematic search of studies published on PubMed, Embase, and Web of Science up to June 1, 2019. We used Stata 15 and Review Manager 5.3 for calculations and statistical analysis.

**Results:**

To evaluate the diagnostic value of TA for BC, we performed a meta-analysis on 22 studies, with a total of 2,867 individuals, including sensitivity, specificity, positive and negative likelihood ratio (PLR, NLR), diagnostic odds ratio (DOR), and 95% confidence intervals (CIs). The pooled parameters were calculated from all studies, and we found a sensitivity of 0.79 (95% CI: 0.72–0.84), a specificity of 0.91 (95% CI: 0.87–0.94), a PLR of 8.91 (95% CI: 5.91–13.43), an NLR of 0.24 (95% CI: 0.15–0.37), a DOR of 37.90 (95% CI: 23.32–61.59), and an AUC of 0.92 (95% CI: 0.90–0.94). We also conducted a subgroup analysis based on the different stages and grades of BC. Results from the subgroup analysis showed that there was no significant difference in TA in either high and low stages of BC, but that low-grade tumors had a lower TA than high-grade tumours.

**Conclusions:**

TA can be used as a potential biomarker for the diagnosis of bladder cancer with its high specificity. Rigorous and high-quality prospective studies are required to verify our conclusion.

## Background

Bladder cancer (BC) is a malignant tumor with very high invasiveness and is one of the ten most common cancer types occurring in both males and females (1, 2). BC can generally be identified using pain-free methods such as macroscopic hematuria or microscopic hematuria, but these methods usually lead to a poor prognosis (3).

Due to the lack of specific clinical symptoms in BC patients, early diagnosis has a great impact on treatment and prognosis (4). Generally, urine cytology, histology, and cystoscopy are the most common methods for diagnosis of BC (5). Biopsy *via* cystoscope for pathological diagnosis is the gold standard for the diagnosis of bladder cancer. Its intuitive characteristics are quite reliable for the diagnosis of BC, but this invasive operation will bring great pain to patients, and its expensive charges also affect its clinical frequency of use and late follow-up (6). The search for a better, lower-risk, accurate, and easy-to-manage methodology for the diagnosis of BC has been ongoing (7).

Detection of telomerase activity (TA) is a non-invasive and effective auxiliary test for the diagnosis of BC (8). Telomerase is correlated to the maintenance of the telomere length in tumor cells and the infinite division of cells. Telomerase activity is present in tumor cells, but it is not typically detected in the normal tissues surrounding the tumor (9). Compared with cystoscopy, the detection of TA can be usually performed using a urine or bladder irrigation solution, which greatly reduces the patient’s fear of medical examination and also facilitates follow-up (10).

Non-invasive diagnostic methods have become a popular and emerging field. There are many studies reporting the accuracy of TA in the diagnosis of BC. However, these diagnostic capabilities are reported by different research groups and thus have significant differences between them. The limitations of these studies are sampling errors and confounding factors within the experiment. Taking into account the limitations of single studies, we performed a meta-analysis based on several research samples and used statistical calculations to better understand the diagnostic efficiency of TA in patients with BC. Some studies have previously revealed the relationship between telomere length and various cancers (11). Based on these studies, we further explored the relationship between TA and BC, with the objective of determining the status of telomeres and telomerase activity and their role in BC.

## Methods

### Literature Search and Eligibility Criteria

We systematically retrieved relevant literature from the PubMed, Embase, and Web of Science databases from inception to June 1, 2019. We used TA, BC, and urine as the search terms, and the search language was limited to English. We also searched the relevant references’ directories to avoid missing other relevant documents.

Studies that meet the following requirements were included in our research: patients diagnosed with BC using the gold standard cystoscopy, studies with the diagnostic value of TA reflected in the research article, and studies with sufficient data on true positive (TP), false positive (FP), false negative (FN), and true negative (TN). Duplicate articles, insufficient quality, studies focusing on other diseases, letters, comments, case reports, and editorials were excluded from our analysis. The review process was assessed by two authors, independently.

### Data Extraction

Studies that meet the following requirements can be included in our research: (1) A patient must be pathologically confirmed and diagnosed as bladder cancer. (2) The patient does not have other malignant tumors of the urinary system. (3) The patient did not perform any invasive transurethral procedures before taking the patient’s urine sample or bladder wash. (4) The telomerase activity in urine or washing fluid samples of all patients has been verified by scientific reagents. (5) The original study provided the number of samples that scientifically proved the telomerase activity in urine or bladder irrigation fluid samples: the number of patients or healthy people whose telomerase activity was positive/negative. Duplicate studies, low-quality studies, studies that cannot extract complete trial data, focus on other diseases, letters, comments, editorials, and case reports are excluded. This process was independently retrieved by two authors (LP and JZL).

### Quality Evaluation

We used the Quality Assessment of Diagnostic Accuracy Studies 2 (QUADAS-2) to assess the quality of the included studies. We also used a quantitative method to assess the selected studies. The QUADAS-2 included 14 items (12). Key domains are assessed to determine the risk of bias and applicability. Signaling questions are included to facilitate judgments, with the risk being low if all signaling answer for a domain is ‘yes’, and if the answer to any question is ‘no’ suggesting potential bias exists. Concerns about applicability are determined as ‘low’, ‘high’, or ‘unclear’.

### Statistical Analysis

We used Stata 15 (StataCorp LP, University City, Texas, USA) and Review Manager 5.3 for the statistical analysis. Using a Q test and I^2^ to evaluate the heterogeneity of the study, I^2^>50% improvement was considered as significantly heterogeneous (13). We used a bivariate model to calculate the pooled sensitivity, specificity, positive and negative likelihood ratios (PLRs and NLRs), diagnostic odds ratio (DOR), and the 95% confidence interval (CI) (14). We calculated the area under the receiver operator characteristic curve (SROC, AUC). AUC varied from 0.5 to 1. If the area was equal to 1, then diagnosis had perfect discrimination. If the area was 0.5, then diagnostic ability was considered as poor (15). Deeks funnel plot was used to assess the publication bias, and Fagan plots showed the relationship between the prior probability, the likelihood ratio, and posterior test probability (16). P<0.05 was considered to be statistically significant.

## Results

### Study Selection and Study Characteristics

**Figure 1:**
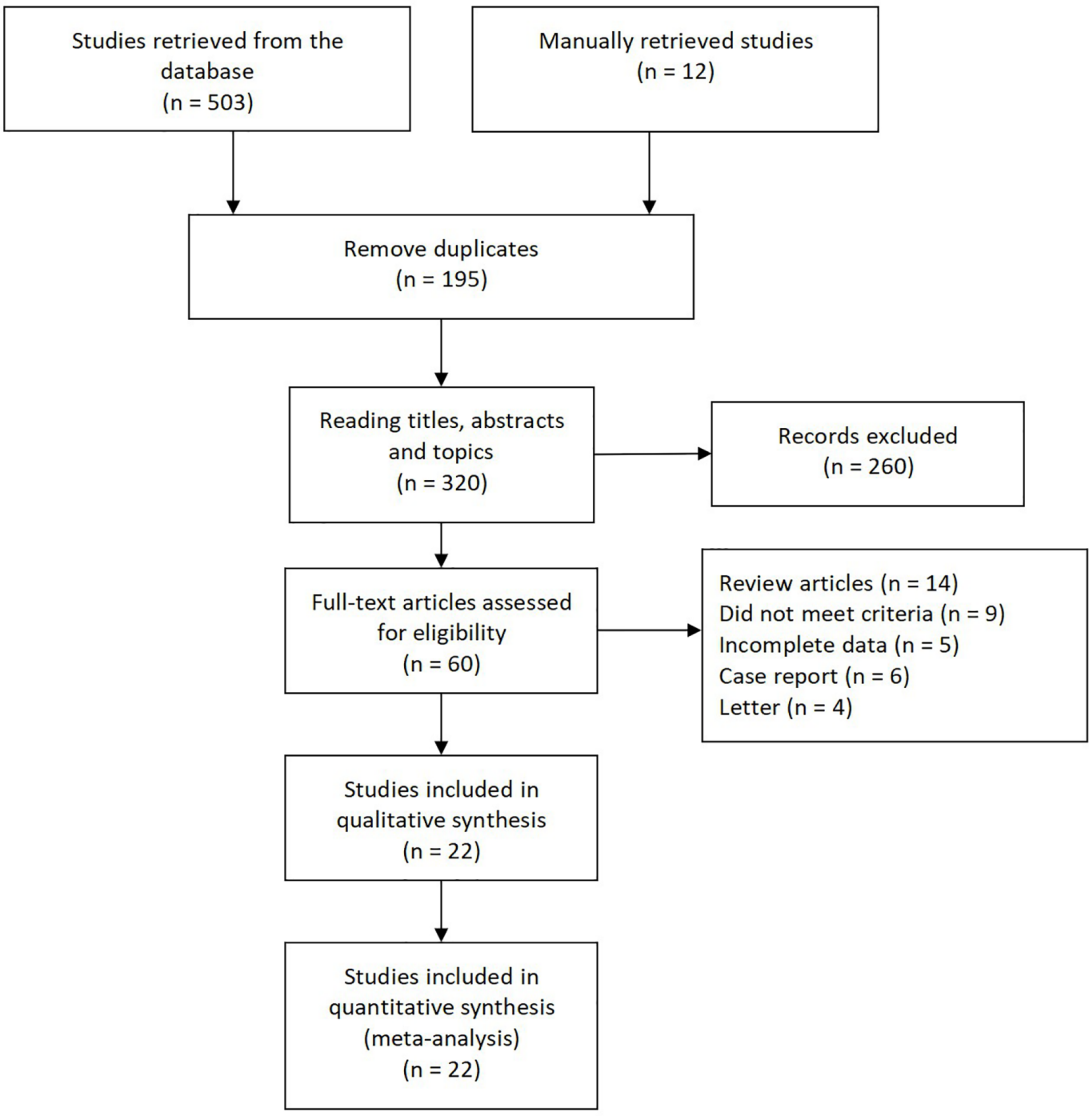
Flow diagram depicting the selection process for all articles found in literature.

**Figure 1** presents the literature search selection process. Initially, we identified a total of 515 studies through the selected databases and manually retrieve. Of these, 195 duplicate records were excluded. After analysis of the title, abstract, and topic, 260 other articles were excluded. 60 articles remained, which were subjected to full text analysis and assessment of eligibility, following which another 38 articles, 14 reviews, six case reports, four Letters, five articles for which data could not be extracted, and nine irrelevant articles were excluded. Finally, we included 22 studies in our qualitative and quantitative analysis (7, 9, 10, 17–35).

In **Table 1**, the characteristics of 22 articles were included in this meta-analysis of TA for BC. The years of these articles are from 1997 to 2010. 2,867 sample individuals from all over the world were included in the study. Most of them were multicenter studies. Sample sizes range from 42 to 185; among 22 studies, five were conducted in Asia (Japan and Israel), six from the United States, 10 from Europe (UK, Germany, Italy and Poland), and an African (Egypt) study. The fourfold table data was presented in **Table 1**.

**Table 1:** Characteristics of the included studies in the meta-analysis.

NO.	Author	Year	Region	Experiment	Control		Study design	Study population	TP	FP	FN	TN	Score of quality
1	Bhuiyan et al. (10)	2003	USA	65	162		Prospective	Multicenter	50	4	15	158	11
2	Bravaccini et al. (7)	2007	Italy	68	144		Prospective	Multicenter	59	49	9	95	10
3	Cassel et al. (9)	2001	Israel	44	29		Retrospective	Single center	37	7	7	22	11
4	Dettlaff et al. (18)	2005	Poland	52	13		Prospective	Single center	47	0	5	13	11
5	Dong et al. (26)	1998	Korea	23	23		Prospective	Single center	22	1	1	22	9
6	Eissa et al. (19)	2007	Egypt	200	115		Prospective	Single center	185	13	15	102	10
7	Guido et al. (17)	1997	USA	37	13		Prospective	Multicenter	13	0	24	13	11
8	Halling et al. (22)	2002	USA	70	80		Prospective	Single center	32	7	38	73	10
9	Kavaler et al. (23)	1998	USA	104	82		Prospective	Single center	88	16	16	66	10
10	Kinoshita et al. (24)	1997	Japan	42	12		Prospective	Single center	23	0	19	12	9
11	Komiya et al. (27)	2009	Japan	75	6		Prospective	Single center	49	1	26	5	10
12	Landman et al. (25)	1998	USA	47	30		Prospective	Single center	38	6	9	24	11
13	Okumura et al. (28)	2004	Japan	37	5		Prospective	Multicenter	23	1	14	4	10
14	Ramakuma et al. (29)	1999	UK	57	139		Prospective	Single center	40	2	17	137	12
15	Roberta et al. (20)	2001	Italy	56	50		Retrospective	Single center	42	9	14	41	10
16	Saad et al. (30)	2002	UK	37	68		Prospective	Single center	26	5	5	63	10
17	Sanchini et al. (31)	2005	Italy	134	84		Prospective	Multicenter	121	10	13	74	11
18	Siracusano et al. (32)	2005	Italy	153	52		Retrospective	Single center	139	11	14	41	9
19	Stefania et al. (21)	2000	Italy	33	20		Prospective	Single center	27	2	6	18	10
20	Steffen et al. (33)	2005	Germany	94	160		Prospective	Single center	70	24	24	136	10
21	Yokota et al. (34)	1998	Japan	29	20		Prospective	Multicenter	25	0	4	20	11
22	Yoshida et al. (35)	1997	UK	26	83		Prospective	Single center	16	3	10	80	12

### Quality Assessment

**Table 1** lists the quality scores for each study. Each article scored 11 points or higher. According to the QUADAS-2 scoring standard, 18 studies were classified with a middle to high score.

### Pooled Diagnostic Values

**Figure 2:**
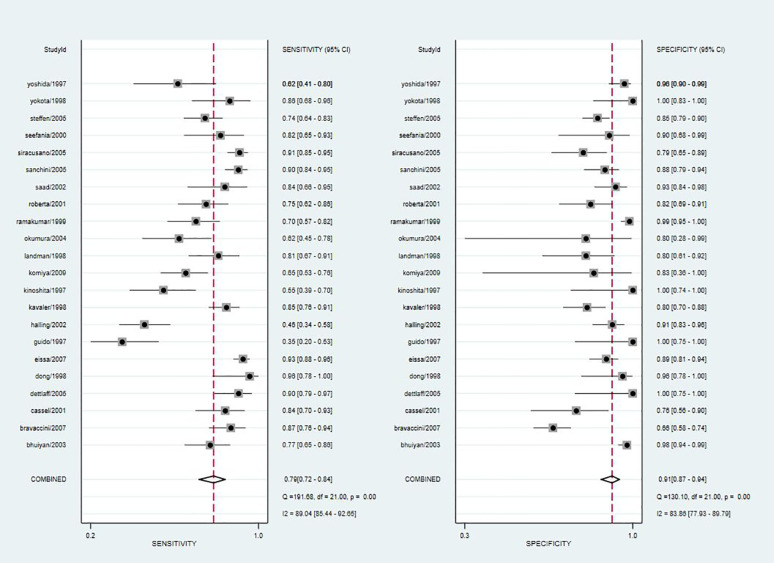
Forest plot of pooled sensitivity and specificity of telomerase activity for bladder cancer.

**Figure 3:**
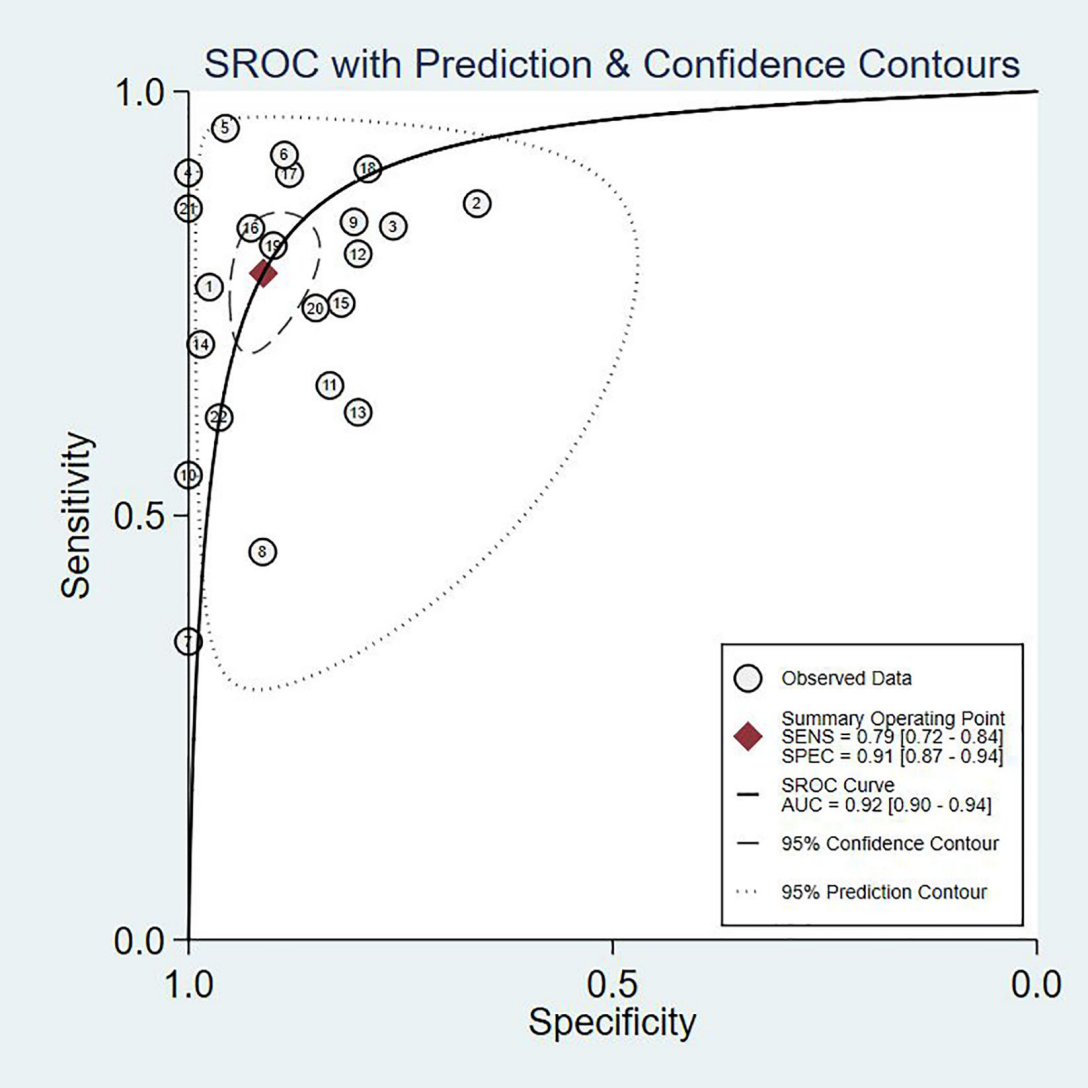
The SROC curve of telomerase activity for bladder cancer.

**Figure 4:**
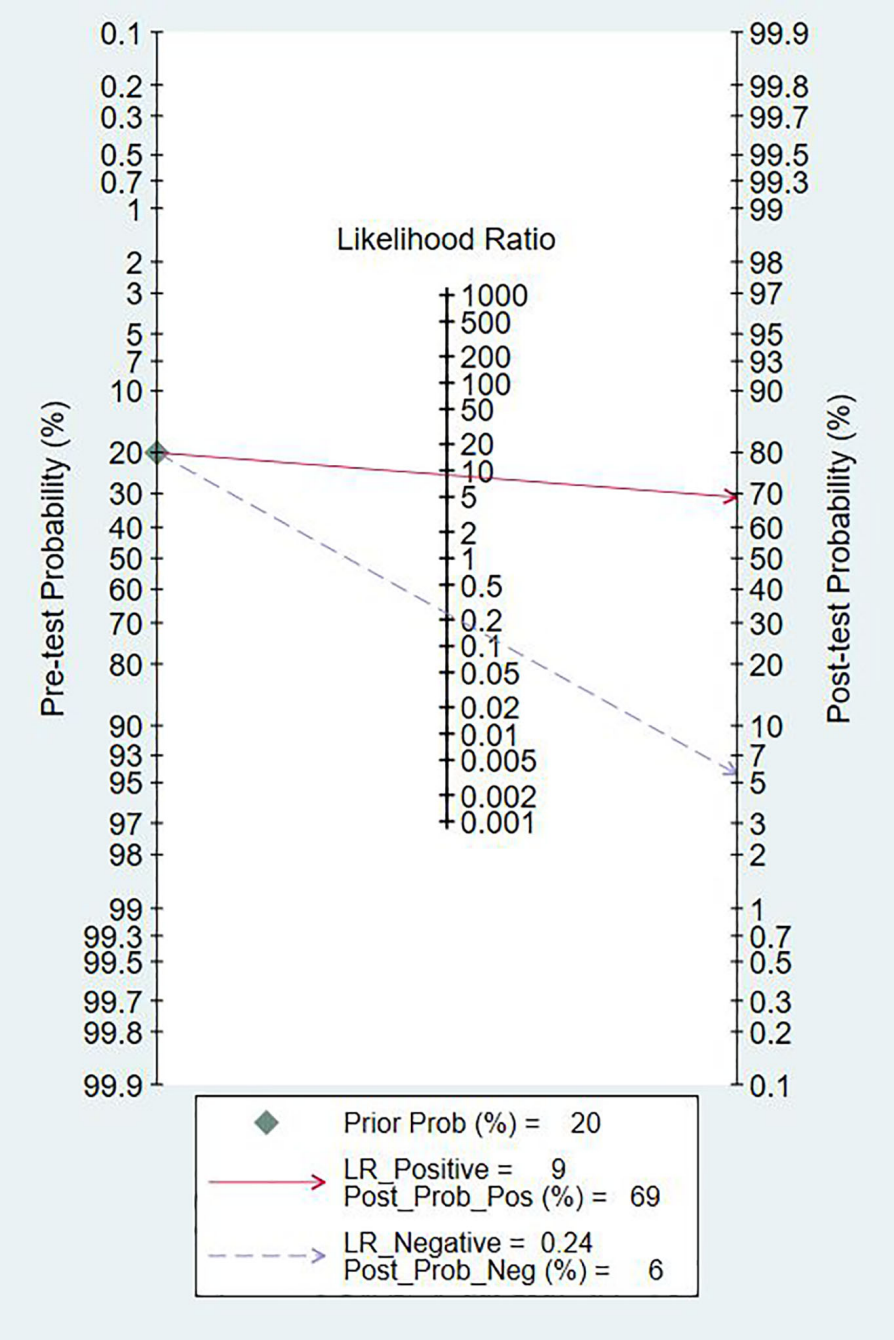
Fagan diagram evaluating the overall diagnostic value of telomerase activity for bladder cancer.

Since the value of I^2^ was greater than 50%, the random effects model was used to combine sensitivity and specificity. The diagnostic value of TA for the detection of BC is shown in **Table 2**. The overall sensitivity and specificity were recorded as 0.79 (95% CI: 0.72–0.84) and 0.91 (95% CI: 0.87–0.94, **Figure 2**), respectively. The Youden Index was 0.7. The pooled PLR was 8.91 (95% CI: 5.91–13.43), NLR was 0.24 (95% CI: 0.15–0.37), and DOR was 37.90 (95% CI: 23.32–61.59). The overall SROC curve is shown in **Figure 3**, with an AUC of 0.92 (95% CI: 0.90–0.94). The Fagan plot is shown in **Figure 4**. The prior probability was 20%, and the post-test probability was 69% for LR-positive and 6% for LR-negative. The diagnostic accuracy for detecting TA in BC was found to be satisfied.

**Table 2:** Summary estimated of diagnostic performance of telomerase activity for bladder cancer.

Category	SEN^1^ (95%CI)	SPE^2^ (95%CI)	PLR^3^ (95%CI)	NLR^4^ (95%CI)	DOR^5^ (95%CI)	AUC^6^ (95%CI)
Overall	0.79(0.65–0.86	0.98(0.94–0.99)	8.91(5.91–13.43)	0.24(0.15–0.37)	37.90(23.32–61.59)	0.92(0.90–0.94)

^1^SEN, Sensitivity; ^2^SPE, Specificity; ^3^PLR, Positive Likelihood Ratios; ^4^NLR, Negative Likelihood Ratios; ^5^DOR, Diagnostic Odds Ratios; ^6^AUC, Area under the curve.

### Subgroup Analyses

**Figure 5:**
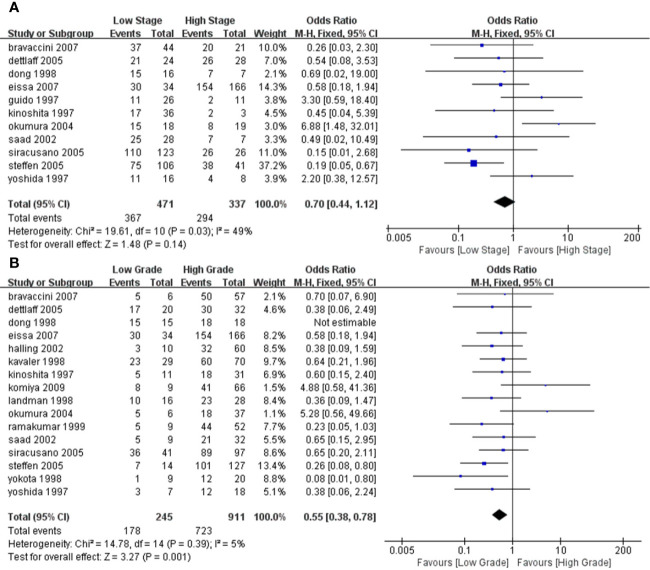
Forest plot depicting the pooled stages and grades for telomerase activity for bladder cancer **(A)**. Forest plot for different stages **(B)**; Forest plot for different grades.

We performed a subgroup analysis of TA based on different stages and grades of the tumors. We specified Tis, Ta, or T1 for a low stage tumor, and T2 and above for a high stage tumor. Similarly, we specified that grade 1 is a low-grade tumor and grades 2–3 is a high-grade tumor. According to the results of the heterogeneity test, we used a fixed model for meta-analysis for both grade and stage, and the results and forest map are shown in **Figure 5**. In our comparison of the different subgroups, the P value was >0.05, suggesting that there was no significant difference in TA in either the high or low stages of a tumor (**Figure 5**). When comparing the different grades, we observed a P=0.001, suggesting that low-grade tumors have lower TA than high-grade tumors.

### Publication Bias

**Figure 6:**
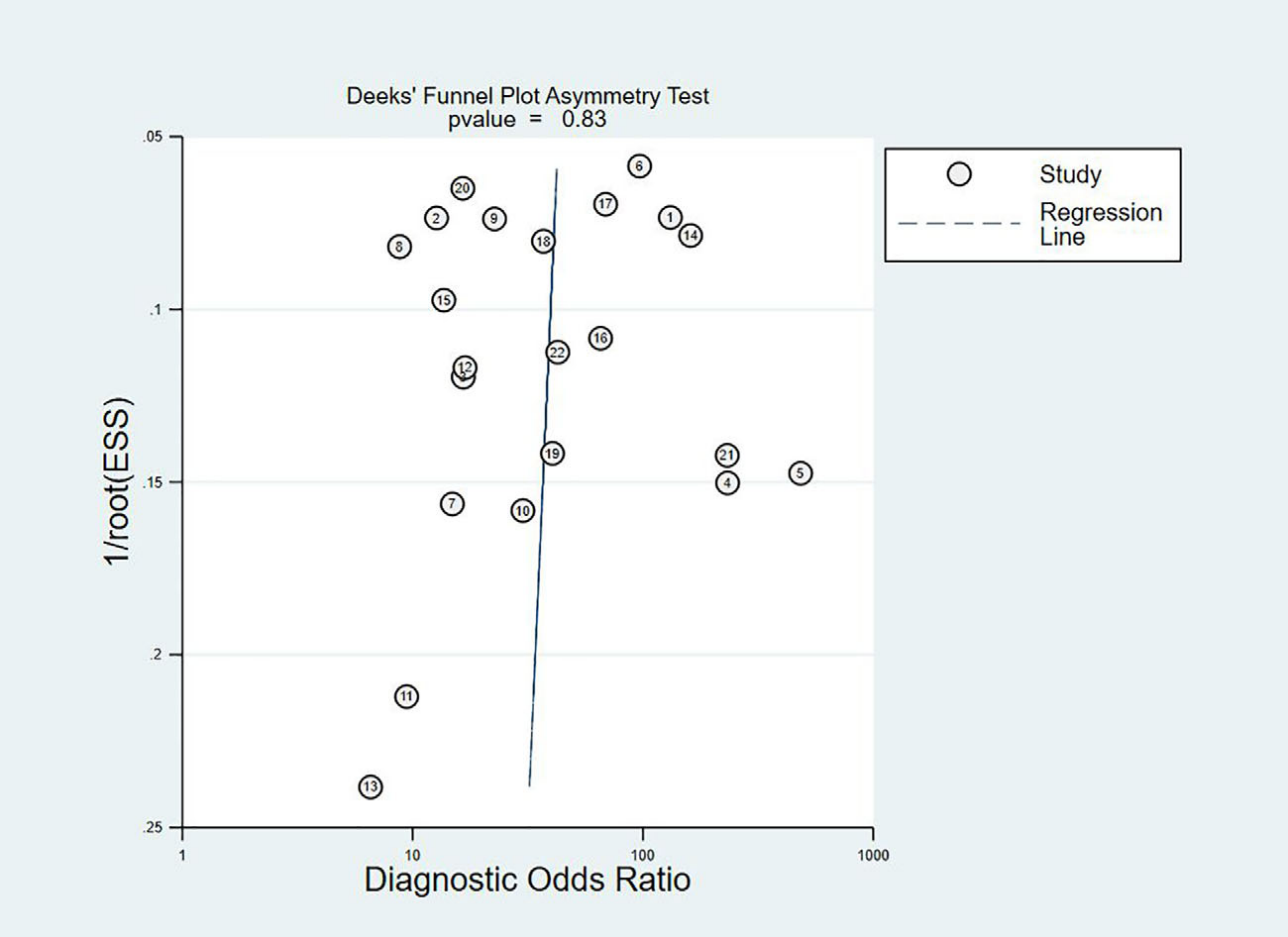
Deek’s funnel plot to evaluate the publication bias.

The Deeks plot showed there was no publication bias (P = 0.83, **Figure 6**).

### Heterogeneity and Sensitivity Analysis

According to the results of the forest plot, the heterogeneity of TA was high in both sensitivity (I^2^ = 89.04%) and specificity (I^2^ = 83.86%). Due to the obvious heterogeneity among the studies, the random effects model was implemented in the calculation and statistics of the combined results to obtain a relatively conservative confidence interval.

### Meta-Regression Analysis

**Figure 7:**
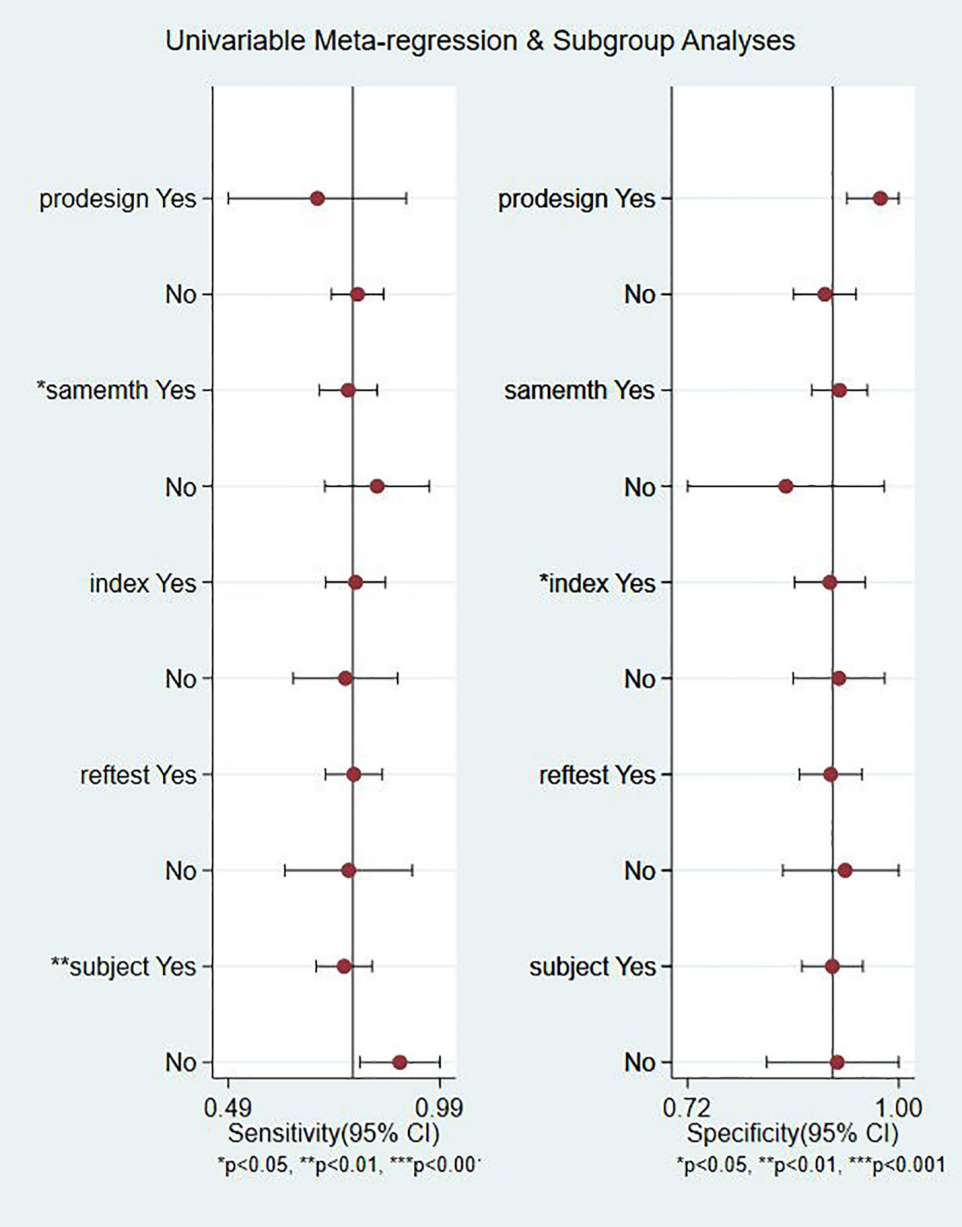
Meta regression and subgroup analysis.

Meta-regression analyses were performed on study design (predesign), gold standard selection, and description (samemth and reftest), diagnostic test to be evaluated (index), and patient characteristics (subject). It can be seen from the forest plot that in the 22 studies we included, gold standard selection, diagnostic test evaluation, and patient characteristics have statistically significant effects on the heterogeneity of sensitivity and specificity (P < 0.05, **Figure 7**).

## Discussion

To our knowledge, this is the first meta-analysis of the diagnostic efficacy for TA in BC. We found that TA is optimal among various other indicators and proved to be an excellent diagnostic.

BC, as a malignant tumor with high morbidity and mortality, has received wide attention, both for its diagnosis and treatment (36). As accepted, cystoscopy has been the gold standard for the diagnosis of BC. Despite its reliability, as an invasive examination, it is performed under local anesthesia, causing strong discomfort to patients (7). A simpler diagnostic would be preferable and would also minimize the damage caused by the examination. There is a shift in the continuous detection and development of BC test methods from macro to micro, and role of markers in urine is being explored for the detection and diagnosis of BC (18, 19).

Telomeres are composed of repeated gene sequences and related proteins. Their main role is to avoid end-to-end fusion and nuclear cleavage during chromosome division (18). Telomerase reverse telomeres shortening during cell division. This is one of the essential processes for the permanent life of tumor cells (9). We hypothesized that in tumor cells, telomerase activity would be higher than in normal cells. Many scholars have studied the relationship between TA and BC, but due to limitations in detection technology and in sample size, the conclusions were so far inconsistent. We integrated and analyzed research done by other authors and included a sample group large enough for performance of meta-analysis, aiming at comprehensive evaluation of the diagnostic validity for looking into TA in BC, with a goal of providing better guidance for clinical practice.

A number of studies have shown that the sensitivity of the telomerase assay for urothelial carcinoma is lower in voided urine specimens than in bladder washings (17, 22, 24). However, urine is easier to obtain than bladder washings and also easier to collect from the patient’s perspective. In our meta-analysis, the overall sensitivity was 0.79 (95% CI: 0.72–0.84), the specificity was 0.91 (95% CI: 0.87–0.94), and the Youden index was 0.7. AUC was 0.92 (95% CI: 0.90–0.94), which was in line with our initial predictions. Using these composite indicators, we showed that TA could be a good and accurate indicator for the diagnosis of BC. A diagnostic test can typically be considered to have a high value when both sensitivity and specificity are >0.7. In this study, consistently with our predictions, the sensitivity results reached this value for 16 articles, again indicating the superiority of TA in the diagnosis of BC. However, the sensitivity values provided in the other two studies were significantly lower (17, 22). The reason for this analysis was that, due to the technical level of the test, the sample size and bias between the samples might have led to different final results. In relation to the specificity results, 21 of the included studies reached 0.7 or higher, showing that the results were not significantly different between the studies and confirming our hypothesis and indicating the excellent specificity of TA for the diagnosis of BC. The higher the value of DOR, the better the diagnostic ability for the selected method. In our study, the DOR value was 37.90 (95% CI: 23.32–61.59), suggesting that the overall accuracy was high. The overall PLR value was 8.91 (95% CI: 5.91–13.43), suggesting that patients with BC have a TA 8.91 times higher than normal, and a total NLR of 0.24 (95% CI: 0.15–0.37), meaning that normal individuals suffering from BC was of 25%. In the judging criteria, PLR>10, NLR<0.1, the diagnostic efficiency for this method was higher. Taking this aspect into account, we can conclude that the diagnostic efficiency of TA for BC is suboptimal. At the same time, we also noticed that the publication bias shown by Deek’s funnel plot (**Figure 6**) has a P-value of 0.83. This shows that there is no significant publication bias among the studies. However, according to the results of Meta regression analysis, the included studies are not consistent due to gold standard selection, diagnostic test evaluation, and patient characteristics, which may be the direct cause of significant heterogeneity (**Figure 7**).

To investigate the TA relationship between different staging and grading, we performed a subgroup analysis. In terms of staging, we considered Tis, Ta, and T0 as low-stage tumors, while T2–T4 as high stage. When grading, grade 1 was considered a low-grade tumor, and grades 2 and 3 were considered high-grade tumors. Thus, through meta-analysis, the association between them was evaluated. We found there was no absolute difference in TA between high-stage and low-stage tumors (P > 0.05) or between different grades, using meta-analysis. Our results showed that the TA in low-grade tumors was significantly lower than in high-grade tumors (P = 0.001). We believe that this is because the higher the grade, the lower the degree of differentiation, the stronger the invasive ability, and the higher the TA, consistent with results reported by Bravaccini et al (7). Detecting TA is not the only non-invasive method used in the diagnosis of BC, and other markers such as nuclear matrix protein (NMP)-22, bladder tumor antigen (BTA), cytokeratin 20 could also be evaluated. Studies have reported that BTA and cytokeratin 20 are not sensitive markers for low-grade tumors. For grade 1 tumors, the sensitivity of BTA and cytokeratin was 13 and 6%, respectively and NMP-22 had a specificity of 70% in the diagnosis of BC (9, 22, 25). NMP-22 is a quick, point of care test having higher sensitivity. In a diagnostic test that included 380 samples, the sensitivity of NMP-22 was 81.9% but at the cost of specificity of 76.97% (37). According to a meta-analysis that included 22 studies, the diagnostic accuracy of urine cytokeratin 20 for bladder cancer is improved with the progression of tumor stage and grade (38). A high-quality meta-analysis including 57 papers showed that across biomarkers, sensitivities ranged from 0.57 to 0.82 and specificities ranged from 0.74 to 0.88. Urine biomarkers plus cytological assessment are more sensitive, but no more specificitive. It is easy to cause missed diagnosis. For patients with low-stage and low-grade tumors, the accuracy of urine biomarkers is poor (39). Therefore, as individual indicators, these markers may work better than invasive methods, but the diagnostic performance should take into consideration composite indicators.

Cystoscopy biopsy, as an invasive examination method, brings pain to patients to a certain extent. For elderly men with enlarged prostate, cystoscopy is more likely to cause prostate bleeding, pain, infection, and other related complications. Anatomically shorter urethra of female patients solves some obstacles for cystoscope access. If the patient has bladder inflammation or tuberculosis infection, the biopsy forceps for the removal of bladder mucosa tissue will make the incidence of bleeding, infection, severe pain, and other complications higher. Whether the clinician’s judgment on the location of the lesion is accurate also affects whether the patient needs to undergo a second cystoscopy to determine the lesion. At present, the diagnosis of malignant tumors is transitioning from invasive to non-invasive. Finding suitable body fluid/blood biomarkers to improve tumor diagnosis is the current research direction. The continuous improvement of the detection technology of telomerase activity in the urine of patients with bladder cancer also indicates that the research of telomerase is becoming more precise and is expected to be widely used in clinical applications in the future (10, 40).

We followed the PRISM guidelines for our meta-analysis (41). However, at present, our meta-analysis still has some limitations. Firstly, of all the studies included here, most of the research samples were from Europe and the United States, which may skew our research due to racial differences. Secondly, in each group of controlled studies, the patients studied may have presented with other diseases. Since the mechanisms are unknown for these, the interactions between the different diseases may have led to changes in the accuracy of our results. Finally, in the subgroup analysis, we combined the different stages and grades of tumours into a single control. Due to the influence of the original data, it was not able to be detailed enough in different stages and grades. Compared with cystoscopy, although not further clinically applied, TA does have a higher advantage in diagnosing BC with its relatively high sensitivity and non-invasive mode of operation. A larger sample size, tighter design, and longer follow-up randomized controlled trials are also needed to validate.

## Conclusions

Based on current evidence, TA can be used as a potential biomarker for the diagnosis of bladder cancer with its high specificity. However, TA performance is not always satisfactory in terms of sensitivity, which may require repeated testing. The maturity of the testing technology will also affect the false negative rate. Further studies in TA is needed, which is more in line with the concept of non-invasive diagnosis of diseases. Rigorous and high-quality prospective studies are required to verify our conclusion.

## Data Availability Statement

The datasets presented in this study can be found in online repositories. The names of the repository/repositories and accession number(s) can be found in the article/supplementary material.

## Author Contributions

Conceived and designed the experiments: YL. Analyzed the data: LP, JZL, CM. Contributed reagents/materials/analysis: JML, DT, TW, PX, and FG. Wrote the manuscript: LP and JZL. All authors contributed to the article and approved the submitted version.

## Funding

This study was supported by Sichuan Science and Technology Program under Grant number 2020YFS0320; National Natural Science Foundation of China, grant number: 81900617, application code: H0503, relying unit code: 63700708A0140-0268; Sichuan Provincial Health Committee Program of China, grant Number: 20PJ305.

## Conflict of Interest

The authors declare that the research was conducted in the absence of any commercial or financial relationships that could be construed as a potential conflict of interest.

## Abbreviations

BC, Bladder Cancer; TA, Telomerase Activity; QUADAS-2, Quality Assessment of Diagnostic Accuracy Studies 2; TP, True Positives; FP, False Positives; FN, False Negatives; TN, True Negatives; ROC, Receiver Operating Characteristic; SROC, Summary Receiver Operator Characteristic; PLRs, Positive Likelihood Ratios; NLRs, Negative Likelihood Ratios; DORs, Diagnostic Odds Ratios; CI, Confidence Intervals.
